# Reliability and practicability of PSMA-RADS 1.0 for structured reporting of PSMA-PET/CT scans in prostate cancer patients

**DOI:** 10.1007/s00330-023-10083-7

**Published:** 2023-08-25

**Authors:** Freba Grawe, Franziska Blom, Michael Winkelmann, Caroline Burgard, Christine Schmid-Tannwald, Lena M. Unterrainer, Gabriel T. Sheikh, Paulo L. Pfitzinger, Philipp Kazmierczak, Clemens C. Cyran, Jens Ricke, Christian G. Stief, Peter Bartenstein, Johannes Ruebenthaler, Matthias P. Fabritius, Thomas Geyer

**Affiliations:** 1 Department of Radiology, University Hospital, LMU Munich, Marchioninistr. 15, 81377 Munich, Germany; 2 Department of Nuclear Medicine, University Hospital, LMU Munich, 81377 Munich, Germany; 3 Department of Nuclear Medicine, Saarland University Hospital, Kirrberger Str., Geb. 50, 66421 Homburg, Germany; 4 Department of Urology, University Hospital, LMU Munich, 81377 Munich, Germany

**Keywords:** Prostate cancer, PSMA, Positron emission tomography computed tomography

## Abstract

**Objectives:**

As structured reporting is increasingly used in the evaluation of prostate-specific membrane antigen positron emission tomography/computed tomography (PSMA-PET/CT) for prostate cancer, there is a need to assess the reliability of these frameworks. This study aimed to evaluate the intra- and interreader agreement among readers with varying levels of experience using PSMA-RADS 1.0 for interpreting PSMA-PET/CT scans, even when blinded to clinical data, and therefore to determine the feasibility of implementing this reporting system in clinical practice.

**Methods:**

PSMA-PET/CT scans of 103 patients were independently evaluated by 4 readers with different levels of experience according to the reporting and data system (RADS) for PSMA-PET/CT imaging PSMA-RADS 1.0 at 2 time points within 6 weeks. For each scan, a maximum of five target lesions were freely chosen and stratified according to PSMA-RADS 1.0. Overall scan score and compartment-based scores were assessed. Intra- and interreader agreement was determined using the intraclass correlation coefficient (ICC).

**Results:**

PSMA-RADS 1.0 demonstrated excellent interreader agreement for both overall scan scores (ICC ≥ 0.91) and compartment-based scores (ICC ≥ 0.93) across all four readers. The framework showed excellent intrareader agreement for overall scan scores (ICC ≥ 0.86) and compartment-based scores (ICC ≥ 0.95), even among readers with varying levels of experience.

**Conclusions:**

PSMA-RADS 1.0 is a reliable method for assessing PSMA-PET/CT with strong consistency and agreement among readers. It shows great potential for establishing a standard approach to diagnosing and planning treatment for prostate cancer patients, and can be used confidently even by readers with less experience.

**Clinical relevance statement:**

This study underlines that PSMA-RADS 1.0 is a valuable and highly reliable scoring system for PSMA-PET/CT scans of prostate cancer patients and can be used confidently by radiologists with different levels of experience in routine clinical practice.

**Key Points:**

- *PSMA-RADS version 1.0 is a scoring system for PSMA-PET/CT scans. Its reproducibility needs to be analyzed in order to make it applicable to clinical practice.*
- *Excellent interreader and intrareader agreement for overall scan scores and compartment-based scores using PSMA-RADS 1.0 were seen in readers with varying levels of experience.*
- *PSMA-RADS 1.0 is a reliable tool for accurately diagnosing and planning treatment for prostate cancer patients, and can be used confidently in clinical routine.*

## Introduction

Prostate-specific membrane antigen (PSMA) is overexpressed in most prostate cancers (PC) and is used as a target of theranostic radiotracers for diagnosis and therapy [1]. Targeted imaging with positron emission tomography/computed tomography (PET/CT) for staging, stratification for PSMA-directed radioligand therapy, and assessment of treatment response is now widely used in clinical practice at specialized centers [2–5]. Based on the positive VISION trial (additive ^177^Lu-PSMA-617 therapy significantly prolonged progression-free survival and overall survival with preserved quality of life) and the approval of the first targeted radioligand therapy for the treatment of progressive PSMA-positive metastatic castration-resistant PC by the US Food and Drug Administration (FDA) and the European Commission (EC) last year, PSMA therapy and, consequently, diagnostic PET/CT will be increasingly offered in more centers in the near future [6]. Considering this and since the assessment of PSMA-PET/CT is not without pitfalls, accurate and standardized evaluation of PSMA-targeted PET imaging findings is of utmost importance [7]. This ensures the best possible selection of suitable patients for radioligand therapy and optimal therapy monitoring, which ultimately determines the patient’s outcome while preventing unnecessary and costly overtreatment. Furthermore, standardization of PSMA-PET interpretation also helps to improve the reproducibility of data in future clinical trials.

Several reporting frameworks have been proposed for PSMA-PET/CT examinations to support image interpretation including therapy response evaluation, and their use is generally recommended by international guidelines [8–15]. When interpreting PSMA-directed PET/CT scans, the reader must navigate around certain pitfalls, including the normal biodistribution of different PSMA-directed PET radiotracers, the varying uptake of radiotracers in numerous types of both benign and malignant lesions, and resulting false-positive and false-negative findings. Therefore, Rowe et al introduced a reporting and data system (RADS) for PSMA-PET/CT imaging, termed PSMA-RADS version 1.0, which uses a 5-point scale for the classification of every single lesion and the overall report (PSMA-RADS-1, benign; PSMA-RADS-2, likely benign; PSMA-RADS-3, equivocal; PSMA-RADS-4, prostate cancer highly likely; PSMA-RADS-5, prostate cancer almost certainly) [9, 16]. PSMA-RADS 1.0 has shown promising results as a simple and effective way to interpret PSMA-targeted PET imaging findings with high interreader agreement rates even for readers with less experience in reading scans [17, 18]. Considering that in the future more and more PSMA-PET/CT will be performed in clinical routine and the reporting burden will increase significantly, there is still a lack of knowledge about certain interesting sub-issues of PSMA-RADS, such as the intrareader agreement of IR, especially in the absence of clinical data which corresponds to the real clinical workflow in a high-volume imaging center. This study aimed to determine the inter- and intrareader agreement of four blinded readers with different levels of experience using PSMA-RADS 1.0 for the interpretation of PSMA-PET/CTs in PC patients to assess the feasibility of the proposed framework in clinical routine.

## Materials and methods

### Patient characteristics

**Fig. 1:**
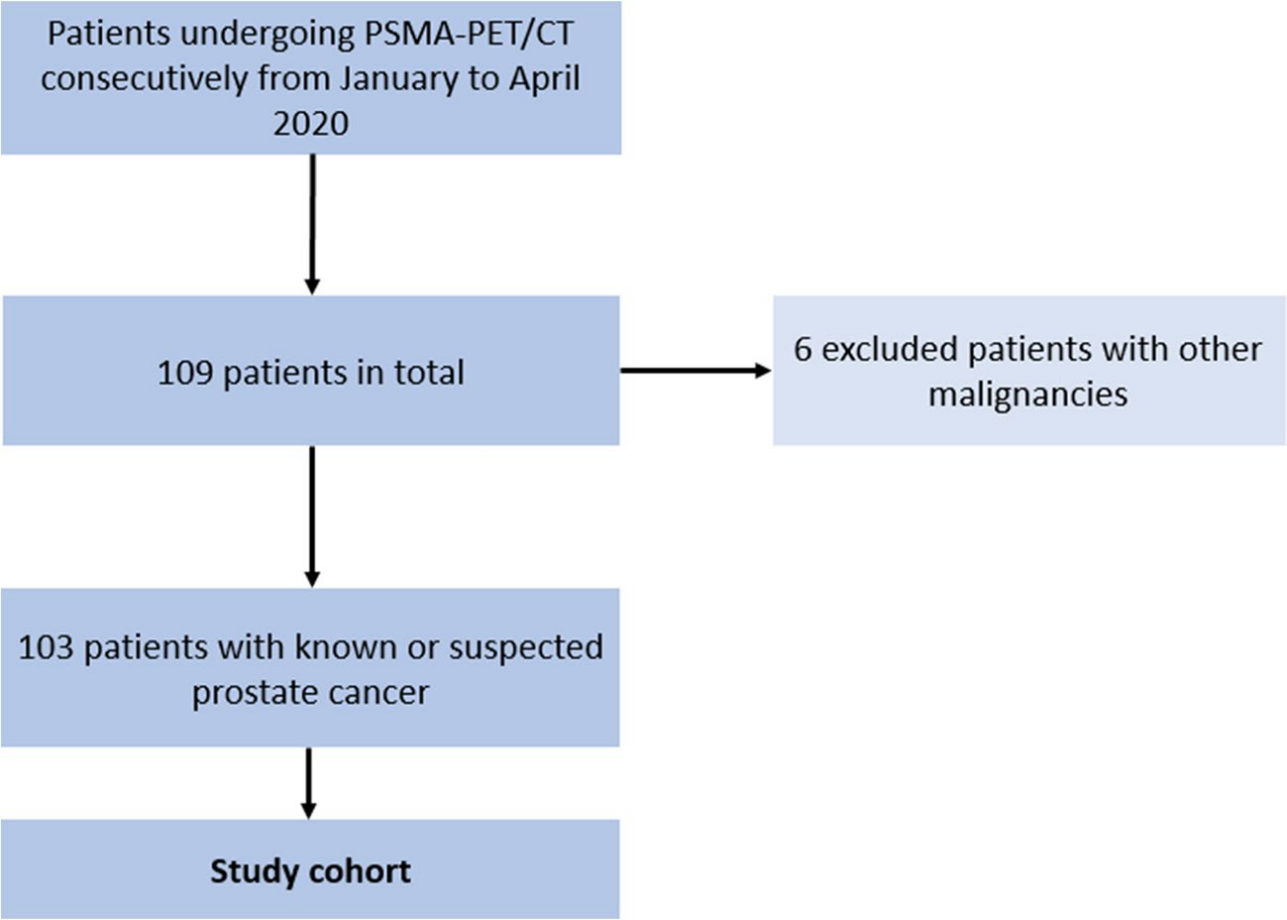
Flowchart illustrating the process of patient selection

A total of 103 patients with known or suspected prostate cancer were retrospectively included in this study. The patients were consecutively selected from the institutional database of all patients who underwent PSMA-targeted PET scans at the institutional department of nuclear medicine from January 2020 to April 2020. Patients with other malignancies than prostate cancer were excluded. The patient selection process is illustrated in Fig. 1. All patient characteristics are displayed in Table 1. The study was approved by the institutional review board (Ethics Committee, Medical Faculty, Ludwig-Maximilians-University Munich; 20-1077; date of approval: 9 December 2020) and conducted according to the principles expressed in the Declaration of Helsinki. Written informed consent was waived by the institutional review board.

**Table 1:** Patient characteristics

Sex	Male	103 (100%)
Age (years)	Mean ± SD (y)	74.0 ± 7.6
	range: 47–90	103 (100%)
	47–60	6 (5.8%)
	61–70	24 (23.3%)
	71–80	52 (50.4%)
	81–90	21 (20.3%)
Indication for scan	Initial diagnosis	11 (10.7%)
	Biochemical recurrence	36 (35%)
	Therapy response assessment	56 (54.4%)
Prior therapies	Total	89 (86.4%)
	Surgery	72 (69%)
	Radiotherapy	38 (36.8%)
	Chemotherapy	4 (3.8%)
	Other	7 (6.7%)
Gleason score (GS)	Mean value ± SD (*n *= 49)	7.7 ± 1
	GS6	1/49 (2.0%)
	GS7	26/49 (53%)
	GS8	10/49 (20.4%)
	GS9	9/49 (18.4%)
	GS10	3/49 (6.1%)
PSA level (ng/ml)	Overall median (*n *= 78)	0.72
	Range	0.03–5000
Additional prostate MRI?	Yes	9/103 (8.7%)
	No	94/103 (91.2%)
Distribution of metastases among patients	Overall positive scan result	73/103 (70.9%)
	Prostate/local recurrence	26 (25.2%)
	Skeleton	60 (58.2%)
	Liver/organs	10 (9.7%)
	Total lymph node (LN)	36 (35.0%)
	*• Axillary*	1 (0.97%)
	*• Hilar/mediastinal*	7 (6.7%)
	*• Retroperitoneal/para-aortic*	15 (14.5%)
	*• Mesenteric*	1 (0.91%)
	*• Iliac*	28 (27.2%)
	*• Inguinal*	6 (5.8%)
Extent of disease	No malignant findings	30/103 (29.1%)
	Solitary malignant lesion	11 (10.7%)
	2–4 malignant lesions	13 (12.6%)
	≥ 5 malignant lesions	49 (47.6%)

*GS*, Gleason score; *LN*, lymph node; *MRI*, magnetic resonance imaging; *PSA*, prostate-specific antigen; *SD*, standard deviation

### Imaging

PSMA-PET/CT scans were acquired on Biograph 64 True-point w/TrueV and Biograph mCT Flow 20-4R PET/CT scanners (Siemens, Healthcare GmbH) and were acquired approximately 60 min after intravenous administration of 248 ± 24 MBq [^18^F]PSMA-1007 (*n *= 100) or [^68^Ga]Ga-PSMA-11 (*n *= 3). Radiolabeling was performed according to good clinical practice [19]. Barring any contraindications, patients were administered 20 mg furosemide along with the tracer injection to avoid bladder activity and to reduce radiation exposure. The radiopharmaceutical was used on an individual patient basis according to the German Pharmaceuticals Act §13(2b). PET was performed from the skull base to the mid-thigh using a Biograph 64 PET/CT scanner or a Biograph mCT scanner (Siemens Healthineers) 60 min after tracer injection PET/CT, and included a diagnostic, contrast-enhanced CT scan (120 kV, 100–400 mAs, dose modulation) of the neck, thorax, abdomen, and pelvis in a portal-venous phase (Imeron 350; 1.5 mL/kg body weight; Bracco Imaging). PET was acquired with 2.5 min per bed position and reconstructed iteratively using TrueX (three iterations, 21 subsets) with Gaussian post-reconstruction smoothing (2 mm full width at half-maximum). Automatic image reconstruction was performed using built-in software. All acquired PET/CT scans were analyzed using dedicated software packages (syngo.via, Siemens Healthcare or Hermes Hybrid Viewer, Hermes Medical Solutions).

### Reading

All scans were independently evaluated by two board-certified radiologists with over 7 years of experience in reading PSMA PET/CT scans (experienced readers, E1 and E2) as well as one radiology resident and one nuclear medicine resident with each about 1 year of experience in reading PSMA PET/CT scans (inexperienced readers, I1 and I2). All readers were masked to clinical data of the patients except for their age.

The interpretation of all images was based on the previously published PSMA-RADS version 1.0 reporting system [9]. Although all readers were familiar with the framework, they received a structured introduction to the PSMA-RADS version 1.0 reporting system and a brief training session by assessing five PSMA PET/CT scans before reading. PSMA-RADS-1 is used for benign lesions characterized by biopsy or pathognomonic finding on anatomic imaging either without abnormal uptake (PSMA-RADS1A) or with abnormal uptake (PSMA-RADS-1B). PSMA-RADS-2 is assigned to a likely benign lesion with low tracer uptake in atypical sites for PC (e.g., axillary lymph nodes). PSMA-RADS-3 is divided into 4 subgroups (A–D) and suggests either further work-up or follow-up imaging to enable final lesion characterization. PSMA-RADS-3A is assigned to lesions with equivocal uptake in soft-tissue site typical of PC involvement (e.g., pelvic or retroperitoneal lymph nodes) and PSMA-RADS-3B for equivocal uptake in a bone lesion not definitive but also not atypical of PC on anatomic imaging (e.g., classic osteoblastic lesion). In these cases, follow-up imaging after 3–6 months is recommended. For PSMA-RADS-3C (intense uptake in site highly atypical of all but advanced stages of PC) and PSMA-RADS-3D (lesion suggestive of malignancy on anatomic imaging but lacking tracer uptake), biopsy is recommended to confirm diagnosis histologically. PSMA-RADS-4 describes lesions with high likelihood of malignancy due to intense tracer uptake but without suspicious corresponding findings on CT imaging.

Biopsy for diagnosis confirmation is not necessarily needed. PSMA-RADS-5 describes intense uptake in site typical of PC with corresponding finding on CT, which is almost certainly malignant. For further analysis, we have subsumed the individual subcategories for a better overview (PSMA-RADS 1–5).

Up to 5 target lesions (TL) were chosen by the readers for each scan. The readers were encouraged to choose the largest lesions or those with the most intense tracer uptake, although the ultimate selection was left to readers individually. Up to 3 lesions of the same organ compartment (lymph nodes, non-lymphatic soft tissue, liver, lung, thyroid, prostate/local recurrence, bone) were allowed. Each TL was evaluated independently using the PSMA-RADS version 1.0 scoring system. An overall PSMA-RADS score was determined as the highest score of any of the TLs. Furthermore, all involved organ compartments were identified.

After a minimum of 4 weeks after the first reading session, all PSMA PET/CT scans were analyzed again by the 4 readers in a different order and blinded to their first reading to achieve a higher number of total evaluated lesions and to assess intrareader agreement.

### Statistical analysis

All calculations were performed using SPSS statistics software (version 25, IBM). Categoric data are displayed as frequency (*n*) and percentage (%). Continuous data are displayed as mean ± standard deviation (SD). The inter- and intrareader agreement was calculated using the intraclass correlation coefficient (ICC) with 95% confidence interval (CI). In line with previous publications based on the model by Cicchetti, agreement was considered poor for ICC < 0.4, fair for ICC between 0.4 and 0.59, good for ICC between 0.6 and 0.74, and excellent for ICC > 0.74 [20]. *p* < 0.05 was considered to indicate statistical significance.

## Results

### Interreader agreement for different compartments

A total of 2092 TL were evaluated by all 4 readers (1083 TL in the first read, 1009 in the second read). Two hundred thirty-eight identical TLs were selected by all 4 readers (118 in the first read, 120 in the second read). In the first read, 47 soft tissue lesions, 48 skeleton lesions, 18 lymph nodes, 1 liver lesion, and 4 prostate/local recurrence lesions were selected identically. In the second read, 48 soft tissue lesions, 50 skeleton lesions, 20 lymph nodes, no liver lesions, and 2 prostate/local recurrence lesions were selected.

The interreader agreement for identically selected TL was excellent with an ICC of 99% both in the first and in the second read. The agreement for ER was slightly higher with ICC of 99% in both reads compared to 97% for IR.

The compartment-based analysis (Table 2) showed excellent agreement for all organs including local recurrence and lymph node metastases, with an ICC of > 93% for all compartments in both the first and the second read.

**Table 2:** Interreader agreement of PSMA-RADS for 4 identical target lesions (TL) among all 4 readers regarding reader types and organ system

Interreader agreement ICC [95%-CI]	Reader type		Organ system					All readers All organs
	ER	IR	Soft tissue	Skeleton	LN	Liver	Prostate/local recurrence	
1st read	0.990 [0.983; 0.994]	0.971 [0.957; 0.960]	1.000 [1.000; 1.000]	0.926 [0.883; 0.955]	0.959 [0.900; 0.984]	N/A	1.000 [1.000; 1.000]	0.992 [0.990; 0.994]
2nd read	0.992 [0.988; 0.995]	0.970 [0.958; 0.979]	1.000 [1.000; 1.000]	0.936 [0.901; 0.961]	0.948 [0.897; 0.977]	N/A	1.000 [1.000; 1.000]	0.992 [0.990; 0.994]

*ER*, experienced reader; *IC*, confidence interval; *IR*, inexperienced reader; *LN*, lymph node; *N/A*, not available

### Interreader agreement for the overall scan score

**Fig. 2:**
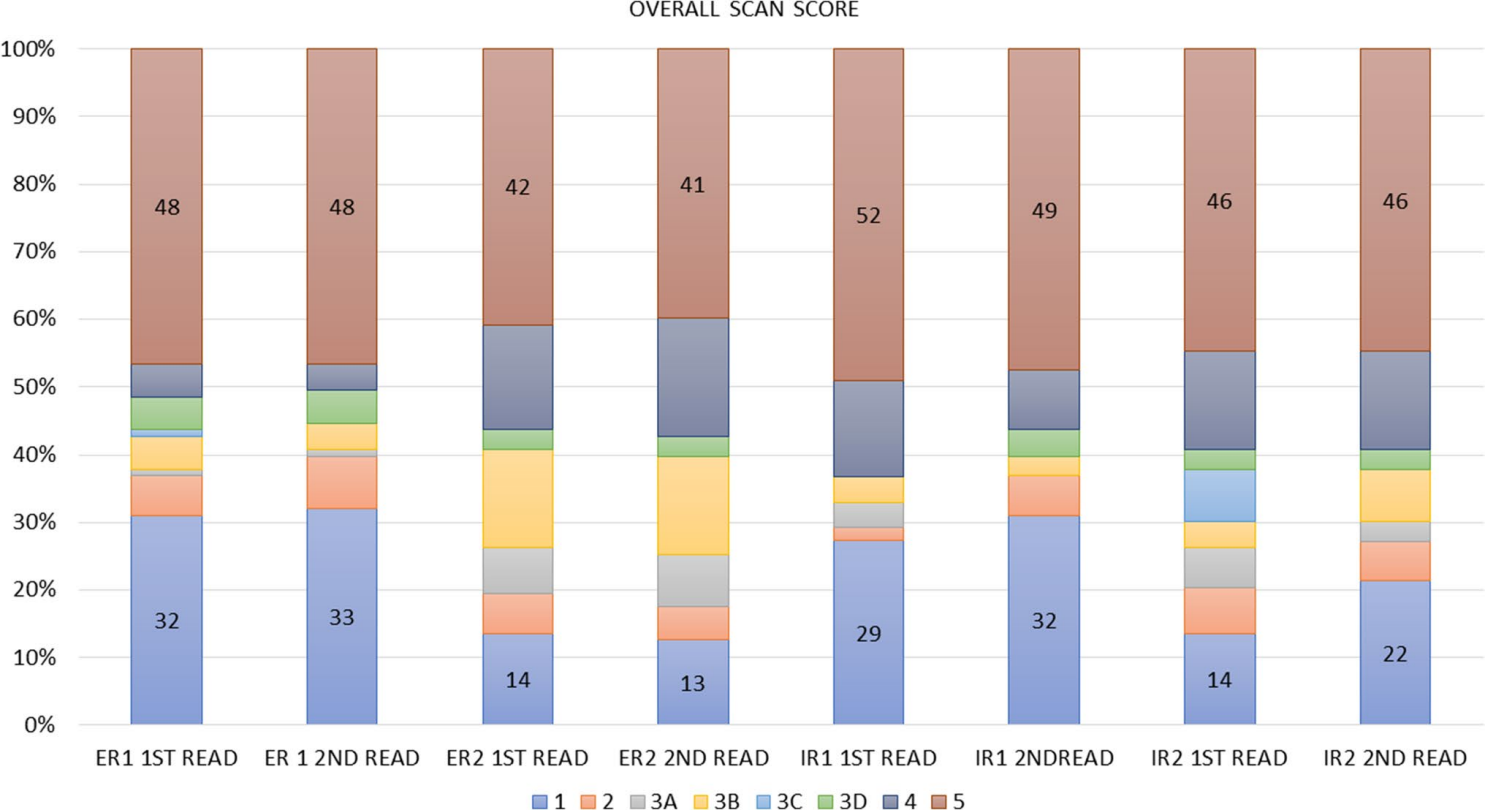
Distribution of PSMA-RADS for the overall scan score of experienced (ER) and inexperienced readers (IR)

The distribution of the overall scan scores of all 4 readers is displayed in Table 3 and Fig. 2. PSMA-RADS 1A and 1B were combined to PSMA-RADS 1. The most frequent scores were PSMA-RADS 5 (372/824, 45.1%) or PSMA-RADS 1 (189/824, 22.9%).

**Table 3:** Overall PSMA-RADS scoring for all 4 readers (103 scans in total)

Distribution of overall PSMA-RADS score of all 4 readers									
PSMA-RADS score		1	2	3A	3B	3C	3D	4	5
1st read	ER1	32	6	1	5	1	5	5	48
	ER2	14	6	7	15	0	3	16	42
	IR1	29	2	4	4	0	0	15	52
	IR2	14	7	6	4	8	3	15	46
2nd read	ER1	33	8	1	4	0	5	4	48
	ER2	13	5	8	15	0	3	18	41
	IR1	32	6	0	3	0	4	9	49
	IR2	22	6	3	8	0	3	15	46

*ER*, experienced reader; *IR*, inexperienced reader

The interreader agreement regarding the overall scan score was excellent with ICC of 92% in the first read and 91% in the second read for all four readers combined. ICC was lower for IR in both reads (75% and 77%) compared to ER (88% and 83%) (Table 4).

**Table 4:** Interreader agreement for the overall scan score among ER and IR

Interreader agreement ICC [95%-CI]	Overall scan score		
	ER	IR	All readers
1st read	0.879 [0.809; 0.922]	0.746 [0.652; 0.829]	0.917 [0.887; 0.940]
2nd read	0.826 [0.725; 0.888]	0.767 [0.657; 0.842]	0.909 [0.877; 0.935]

*ER*, experienced reader; *ICC*, intraclass correlation coefficient; *IR*, inexperienced reader

### Intrareader agreement

**Fig. 3:**
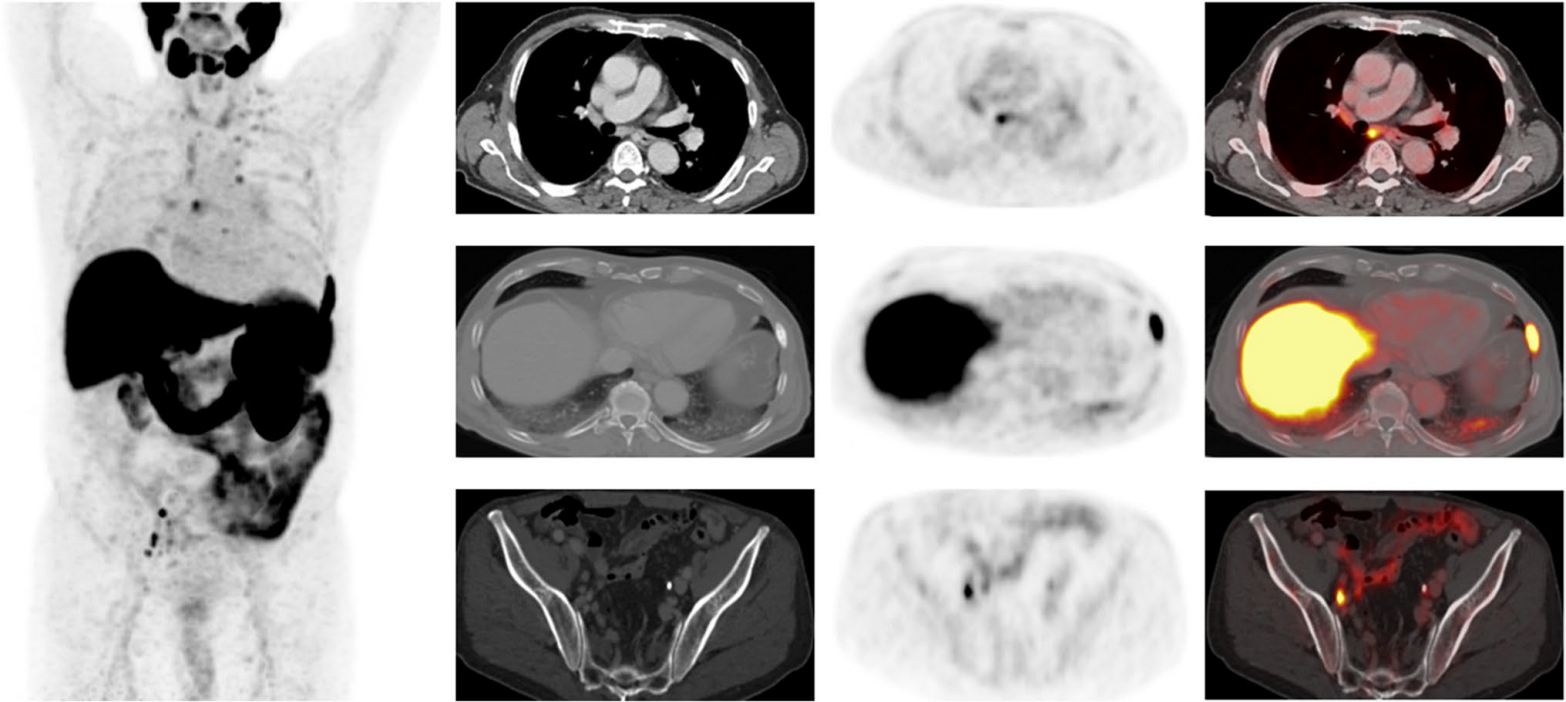
PSMA-PET/CT scan of a 67-year-old patient for diagnostic assessment of histopathologically confirmed prostate cancer. Whole-body maximum-intensity projection (left image) shows several sites of elevated radiotracer uptake in the right lower abdomen and small sites in the mediastinum. Upper row: Axial CT, axial [^18^F]PSMA-PET, and axial fused [^18^F]PSMA-PET/CT reveal mild radiotracer uptake in a subcarinal, not pathologically enlarged mediastinal lymph node. The lesion was classified as PSMA-RADS-4 (likely malignant) by one inexperienced reader, but as PSMA-RADS-2 (likely benign) by both experienced readers. In follow-up images, the lesion showed no suggestive tracer uptake and no progression in size whereas the overall scan showed progressive disease, therefore indicating the subcarinal lymph node to be benign. Middle row: Axial CT (bone window), axial [^18^F]PSMA-PET, and axial fused [^18^F]PSMA-PET/CT show an osteoblastic metastasis in the seventh left rib with intense radiotracer uptake, which was classified PSMA-RADS-5 by all readers. Lower row: Axial CT, axial [^18^F]PSMA-PET, and axial fused [^18^F]PSMA-PET/CT show bilateral iliac lymph nodes without pathological enlargement in CT, but elevated radiotracer uptake. The lesions were called PSMA-RADS-4 by all readers due to lacking definitive malignant findings on CT imaging. The overall scan score was PSMA-RADS-5 for all four readers in both reads, since the metastasis in the seventh left rib showed suggestive radiotracer uptake as well as osteosclerotic changes on CT images

Intrareader agreement for the overall scan score was excellent for all ER and IR (Table 5). Moreover, the organ system–based analysis showed excellent intrareader agreement for all organ systems (Table 6). A case example from the study is shown in Fig. 3.

**Table 5:** Intrareader agreement for the overall scan score among experienced (ER) and inexperienced readers (IR)

Overall scan score				
Reader type	ER1	ER2	IR1	IR2
Intrareader agreement ICC [95%-CI]	0.915 [0.874; 0.942]	0.976 [0.964; 0.984]	0.861 [0.795; 0.906]	0.994 [0.991; 0.996]

*CI*, confidence interval; *ER*, experienced reader; *ICC*, intraclass correlation coefficient; *IR*, inexperienced reader

**Table 6:** Intrareader agreement on organ system–/target lesion–based, scoring among experienced (ER) and inexperienced readers (IR)

	Reader type		Organ system					All readers All organs
	ER	IR	Soft tissue	Skeleton	LN	Liver	Prostate/local recurrence	
Intrareader agreement ICC [95%-CI]	0.9935 [0.991; 0.995]	0.988 [0.983; 0.991]	1.000 [1.000; 1.000]	0.951 [0.920; 0.970]	0.955 [0.906; 0.978]	0.985 [0.853; 0.999]	1.000 [1.000; 1.000]	0.991 [0.987; 0.993]

*CI*, confidence interval; *ER*, experienced reader; *ICC*, intraclass correlation coefficient; *IR*, inexperienced reader; *LN*, lymph node

## Discussion

Our study demonstrates that PSMA-RADS is a reproducible and simple score to assess the extent of the disease in patients with PC. Even IR can quickly and accurately apply the system and achieve a high level of diagnostic confidence.

For the compartment/lesion-based scoring, excellent inter- and intrareader agreement in both the first and second reading session was observed with ICCs > 0.926. The lowest agreement was found for scoring bone lesions. Evaluation of bone lesions represents a possible pitfall due to PSMA-uptake of degenerative alterations and therefore may lead to discrepancies in the assignment of PSMA-RADS scores. The highest agreement was observed for soft tissue lesions and the primary tumor, as these findings are mostly distinct in both CT morphology and PSMA expression. Since the theranostic approach for PC will soon develop into a standardized diagnostic and therapeutic procedure that is more widely used, the accurate assessment of the overall scan score is of paramount importance for selecting eligible patients for radioligand therapy with [^177^Lu]Lu-PSMA-617 [21]. The results support findings of smaller cohorts in terms of overall scan agreement. Werner et al showed good interreader agreement between readers with different levels of experience for PSMA-RADS in 50 PET/CT examinations [22]. Toriihara et al investigated the interreader and for the first time also the intrareader agreement in 57 PET/CT examinations with promising results, but only ERs were included. Moreover, all these ERs were nuclear medicine physicians [23]. We could extend these findings by showing that the reading results are also reproducible with high intrareader agreement among IR. It should also be noted that the readings in our analysis were blinded to clinical data in both sessions, i.e., no prostate-specific antigen (PSA) value was available. The reproducible results, despite the blindness towards clinical data, are consistent with a recent study that showed good interreader agreement in the interpretation of PSMA-PET/CT using PSMA-RADS when readers were blinded to clinical data in one of two reading sessions [24]. However, again, only ERs were included in that study. Our results show that even IR deliver reproducible results in image interpretation despite missing clinical information, which has implications for high throughput in a busy daily clinical setting where patient data cannot always be retrieved at the time of scan interpretation. Nevertheless, one must not forget the importance of clinical parameters, especially PSA, which make an essential contribution to interdisciplinary treatment decisions and monitoring and are also known to correlate with PET/CT findings [25].

Given the fact that PC is the third leading cause of cancer-associated death in men and that the FDA has now approved radioligand therapy for the treatment of progressive PSMA-positive metastatic castration-resistant PC, molecular PSMA imaging is becoming more and more important [21, 26, 27]. As a result, a steady increase in PSMA-targeted scans can be expected in the coming years also in smaller centers with inexperienced physicians. However, recent technological advances, including total-body- and digital PET/CT scanners, will improve sensitivity and subsequently diagnostic performance for the detection of pathological lesions by enhanced spatial resolution, faster time-of-flight, and shorter dead time [28, 29]. A publication by Alberts et al investigated the impact of digital PET/CT, a solid-state detection system, compared to the traditionally analogue PET/CTs with bismuth germinate scintillation crystals coupled with photomultiplier tubes. They reported on a higher detection rate for pathological lesions in [^68^Ga]Ga-PSMA-11 PET/CT for recurrent prostate cancer for digital PET/CT compared with analogue PET/CT without reduced interrater reliability [30]. Since it has been shown that standardized frameworks for PET/CT interpretation are also helpful in the selection and monitoring of ligand therapy [31], our results further encourage even IR at new centers offering these therapies to use PSMA-RADS for PET/CT interpretation, as they can serve as a guide for therapy decisions in multidisciplinary tumor boards when considering ligand therapy. Several other frameworks were also proposed for standardized interpretation of PSMA-PET/CT imaging, such as the EANM Delphi consensus from 2017, which was updated to E-EANM or the “Prostate Cancer Molecular Imaging Standardized Evaluation (PROMISE)” from 2018, which defines molecular imaging TNM (miTNM) regions and subregions for whole-body staging, similar to the pathological/clinical TNM system and the PRIMARY score by Emmett et al assessing patterns of intra-prostatic PSMA [10–13, 32]. However, PSMA-RADS comes with many strengths: The categorical, 5-point scale is similar to other “RADS,” such as BI-RADS for breast lesions in mammography, and therefore familiar to most users and easy to apply. Furthermore, PSMA-RADS falls under the umbrella term molecular imaging reporting and data systems (MI-RADS) and is reciprocal to SSTR-RADS (somatostatin-receptor reporting and data system) for the interpretation of somatostatin-receptor (SSR-PET/CT) [33–36].

Due to the simplicity and good comprehensibility of these frameworks, readers can become acquainted with them in a very short time, and they can apparently be implemented into clinical routine without much effort even in newer centers. Despite excellent results applying PSMA-RADS 1.0 without clinical knowledge, PSA represents an important biomarker for prostate cancer and by the lack of PSA-values in the framework, important information on risk stratification of patients may be missing. In our study, it is noticeable that ERs report PSMA-RADS 1 more frequently than IR. This is an important point to be aware of the possible risk of overdiagnosis. There are several reasons for this: ERs, especially those who specialize in a particular imaging modality, have extensive knowledge and experience in interpreting PSMA-targeted images. They are familiar with the imaging features of benign disease as well as various physiologic and anatomic variations that can mimic suspicious lesions and are therefore better able to classify them more accurately. This knowledge allows experienced investigators to recognize cases that have characteristic benign features and confidently assign them PSMA-RADS 1. In addition, experience increases the investigators’ confidence in their interpretations. ERs may be more comfortable to assign PSMA-RADS 1 when they are confident in their assessment, considering both the imaging features and their clinical experience. IR may exhibit greater caution or uncertainty, resulting in fewer PSMA-RADS 1 assignments. It is worth noting that although experienced readers report PSMA-RADS 1 more frequently, this does not imply that IR are incorrect or less accurate in their assessments. IR may proceed more cautiously, opting for higher PSMA-RADS categories or requesting further examinations to minimize the risk of missing suspicious findings.

There are a few limitations of this study. First, due to the lack of histopathologic correlation of the selected target lesions, potentially false-positive findings are possible. Second, the high agreement rates may not be confirmable for all organ system compartments because, for example, the number of soft tissue lesions selected as target lesions was rather high and those of local recurrences low, as these are often difficult to measure. Furthermore, additional research could assess how well IR perform compared to an optimized benchmark established by either a consensus interpretation from multiple ER or an ER who has access to all clinical information.

## Conclusion

In conclusion, PSMA-RADS 1.0 represents a highly reproducible and accurate system for stratifying PSMA-targeted PET/CT imaging in PC patients with high inter- and intrareader agreement among readers with different levels of experience. The scoring system is a useful tool to simplify and improve the management of PC patients in clinical practice and should be used also by IR without apprehension.

## Abbreviations

CI: Confidence interval
CT: Computed tomography
ER: Experienced reader
FDA: Food and Drug Administration
ICC: Intraclass correlation coefficient
IR: Inexperienced reader
kV: Kilovolt
LN: Lymph node
mAs: Milliampere-seconds
mg: Milligram
PET: Positron emission tomography/computed tomography
PSMA: Prostate-specific membrane antigen
RADS: Reporting and data system
SD: Standard deviation
SSTR: Somatostatin receptor
TL: Target lesion

## Declarations

### Guarantor

The scientific guarantor of this publication is Dr. Thomas Geyer.

### Statistics and biometry

Our institutional statistician Regina Schinner was involved in the statistical analysis.

### Study subjects or cohorts overlap:

No subject subjects or cohort overlap reported.

### Methodology:

- retrospective study
- experimental study
- single-centre
